# Exogenous polyunsaturated fatty acids (PUFAs) promote changes in growth, phospholipid composition, membrane permeability and virulence phenotypes in *Escherichia coli*

**DOI:** 10.1186/s12866-020-01988-0

**Published:** 2020-10-12

**Authors:** Joshua L. Herndon, Rachel E. Peters, Rachel N. Hofer, Timothy B. Simmons, Steven J. Symes, David K. Giles

**Affiliations:** 1 Department of Biology, Geology, and Environmental Science, The University of Tennessee at Chattanooga, Chattanooga, TN USA; 2 Department of Chemistry and Physics, The University of Tennessee at Chattanooga, Chattanooga, TN USA

**Keywords:** *Escherichia coli*, Polyunsaturated fatty acids (PUFAs), Phospholipids, Antimicrobial peptides, Biofilm, Motility

## Abstract

**Background:**

The utilization of exogenous fatty acids by Gram-negative bacteria has been linked to many cellular processes, including fatty acid oxidation for metabolic gain, assimilation into membrane phospholipids, and control of phenotypes associated with virulence. The expanded fatty acid handling capabilities have been demonstrated in several bacteria of medical importance; however, a survey of the polyunsaturated fatty acid responses in the model organism *Escherichia coli* has not been performed. The current study examined the impacts of exogenous fatty acids on *E. coli*.

**Results:**

All PUFAs elicited higher overall growth, with several fatty acids supporting growth as sole carbon sources. Most PUFAs were incorporated into membrane phospholipids as determined by Ultra performance liquid chromatography-mass spectrometry, whereas membrane permeability was variably affected as measured by two separate dye uptake assays. Biofilm formation, swimming motility and antimicrobial peptide resistance were altered in the presence of PUFAs, with arachidonic and docosahexaenoic acids eliciting strong alteration to these phenotypes.

**Conclusions:**

The findings herein add *E. coli* to the growing list of Gram-negative bacteria with broader capabilities for utilizing and responding to exogenous fatty acids. Understanding bacterial responses to PUFAs may lead to microbial behavioral control regimens for disease prevention.

## Background

An emerging body of evidence has highlighted the expanded fatty acid handling characteristics of Gram-negative bacteria. The impacts of fatty acids acquired from growth media include phospholipid remodeling and phenotypes affecting growth and virulence [1–4]. The expanded utility of fatty acids magnifies the relevance of environmental adaptation for bacteria, especially those that oscillate between host and aquatic niches. While the membrane phospholipid modifications and behavioral responses (biofilm formation, motility) of several gammaproteobacteria have been demonstrated, an examination of *Escherichia coli* has not been performed. *E. coli*, regarded as the Gram-negative model organism for bacteria due to its historical prevalence and genetic tractability, has consequently become one of the most well-understood organisms in the world. While the process of bacterial transport of exogenous fatty acids was characterized in *E. coli* [5–7], the range of fatty acids that can be utilized (and for what purposes) has not been investigated.

Although pathogenic strains of *E. coli* do exist, most *E. coli* strains are naturally found within the intestinal tract of humans and other mammals. Pathogenic *E. coli* strains may be separated into two broad categories: those that cause intestinal infections and those that cause extraintestinal infections. Intestinal infections typically result in severe diarrhea, while extraintestinal infections may manifest as urinary tract infections, meningitis, and septicaemia [8]. Previous research has shown that some urinary tract isolates of *E. coli* displayed resistance to at least three of the following antibiotics: ampicillin, cephalothin, ciprofloxacin, nitrofurantoin, and trimethoprim-sulfamethoxazole, allowing the isolates to be considered multi-drug resistant (MDR) [9].

*E. coli* earns its designation among the ESKAPE pathogens by being the leading cause of nosocomial and community-acquired urinary tract infections, coupled with the emergence of MDR strains becoming more prevalent over the last few decades partially due to the selective pressure exerted by antibiotic use [10, 11]. Therefore, counteracting the rise of MDR bacteria is one of the most urgent challenges in the healthcare industry and has necessitated the development of novel treatments [12]. The recently discovered phenomena involving fatty acid responsive behavior in Gram-negative bacteria pose an intriguing therapeutic line of inquiry for exogenous fatty acids.

The current study investigates the physiological and behavioral responses to exogenous fatty acids in *E. coli*. In Gram-negative bacteria, the uptake and assimilation of fatty acids is initiated by the outer membrane transporter FadL which delivers exogenous fatty acids to the periplasm where they can be activated at the inner membrane by the acyl-coenzyme A synthase FadD [13]. Finally, the acyl-CoA is destined for one of two known fates: degradation via the ß-oxidation pathway or assimilation into membrane phospholipids by membrane acyltransferases [14]. A third possibility is recognition of fatty acids as signaling molecules, presumably at the cytosolic membrane to activate a two-component system for behavioral response (eg, motility, biofilm) [15] or via generation of second messengers to regulate homeostasis [16].

The overall goal of this research was to investigate the capability of *E. coli* to assimilate exogenous PUFAs into its membrane phospholipids and to determine the impact that these modifications have on its growth, permeability and virulence phenotypes. We show by Ultra performance liquid chromatography/electrospray ionization-mass spectrometry (UPLC/ESI-MS) analyses that *E. coli* assimilates most fatty acids tested into membrane phospholipids. Growth assays demonstrated *E. coli*’s capacity to use many of the tested PUFAs as a sole carbon source, while crystal violet and ethidium bromide assays portrayed changes in the membrane permeability of PUFA-exposed compared to nonexposed cells. Moreover, the propensity for biofilm formation and degree of motility were found to be influenced by PUFA exposure. Remarkably, some fatty acids altered the resistance of PUFA-exposed *E. coli* to the cationic antimicrobial peptides polymyxin B and colistin.

## Results

### Exogenous fatty acid growth characteristics in *E. coli*

**Fig. 1:**
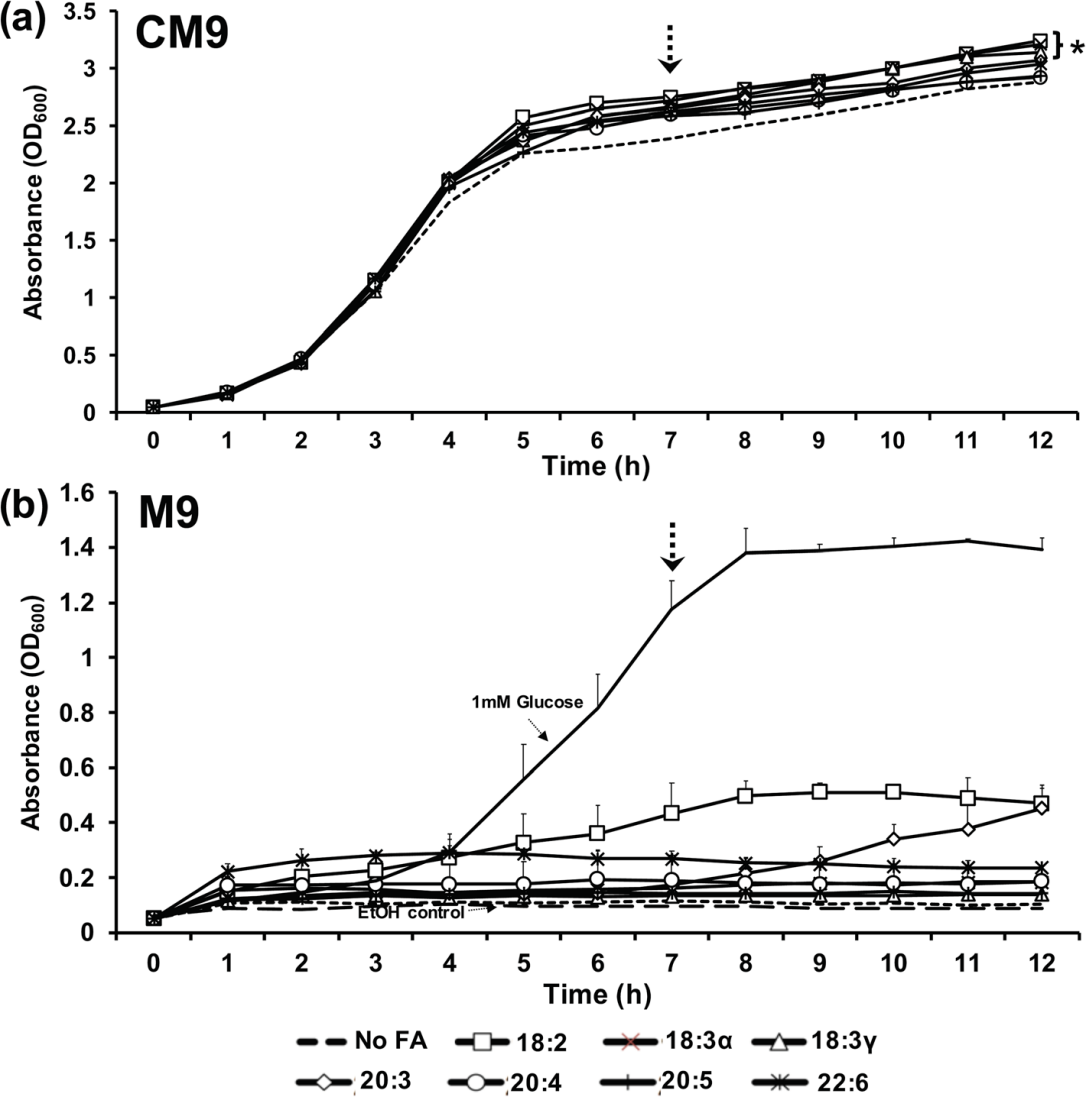
Growth patterns of *E. coli* in minimal media supplemented with exogenous PUFAs as additional carbon sources and as sole carbon sources. **a** Bacteria were grown in CM9 supplemented with 300 μM of individual PUFAs at 37 °C for 12 h. **b** Bacteria were grown in glucose-deficient M9 media supplemented with 1 mM of individual fatty acids as sole carbon sources at 37 °C for 12 h. An ethanol control (67 μM) is included to account for the highest volume of ethanol-dissolved fatty acid supplemented. Graphs represent averages and standard deviations of 3 independent experiments. All standard deviations not visualized by error bars are < 0.13 (**a**) or < 0.03 (**b**). Asterisk indicates significance (*p* < 0.04) calculated using Student’s T-test (2-tail, 2 sample equal variance) comparing growth from hours 6–12 versus control (No FA) sample. Arrows indicate time at which CFU/ml was determined (see Supplemental Table 1)

In Gram-negative bacteria, several studies have reported that supplementation of growth media with unsaturated fatty acids augments growth in logarithmic and stationary phases [1, 2, 4, 17]. When *E. coli* was grown in CM9 in the presence of 300 μM of individual PUFAs, elevated growth (as compared with control) was observed over the course of 12 h (Fig. 1a). Between 6 and 12 h, three fatty acids (18:2, 18:3α, and 18:3γ) correlated with statistically significant (*p* < 0.04) higher growth, as determined by a two-tailed, two sample equal variance t-test.

To evaluate the growth of *E. coli* on fatty acids as a sole carbon source, the bacteria were supplemented with 1 mM of each fatty acid in M9 minimal media (no glucose) and growth was measured for 12 h. Linoleic acid and docosahexaenoic acid caused noticeable growth above other fatty acids, while dihomo-gamma-linolenic acid caused appreciable growth in the latter hours only (Fig. 1b). Viability and purity of cultures was confirmed by plating for colonial growth at hour 12. CFU determination was performed at hour 7. All fatty acid-supplemented samples yielded higher CFU/ml (Supplemental Table 1).

### *E. coli* incorporates exogenous PUFAs into its phospholipids

**Fig. 2:**
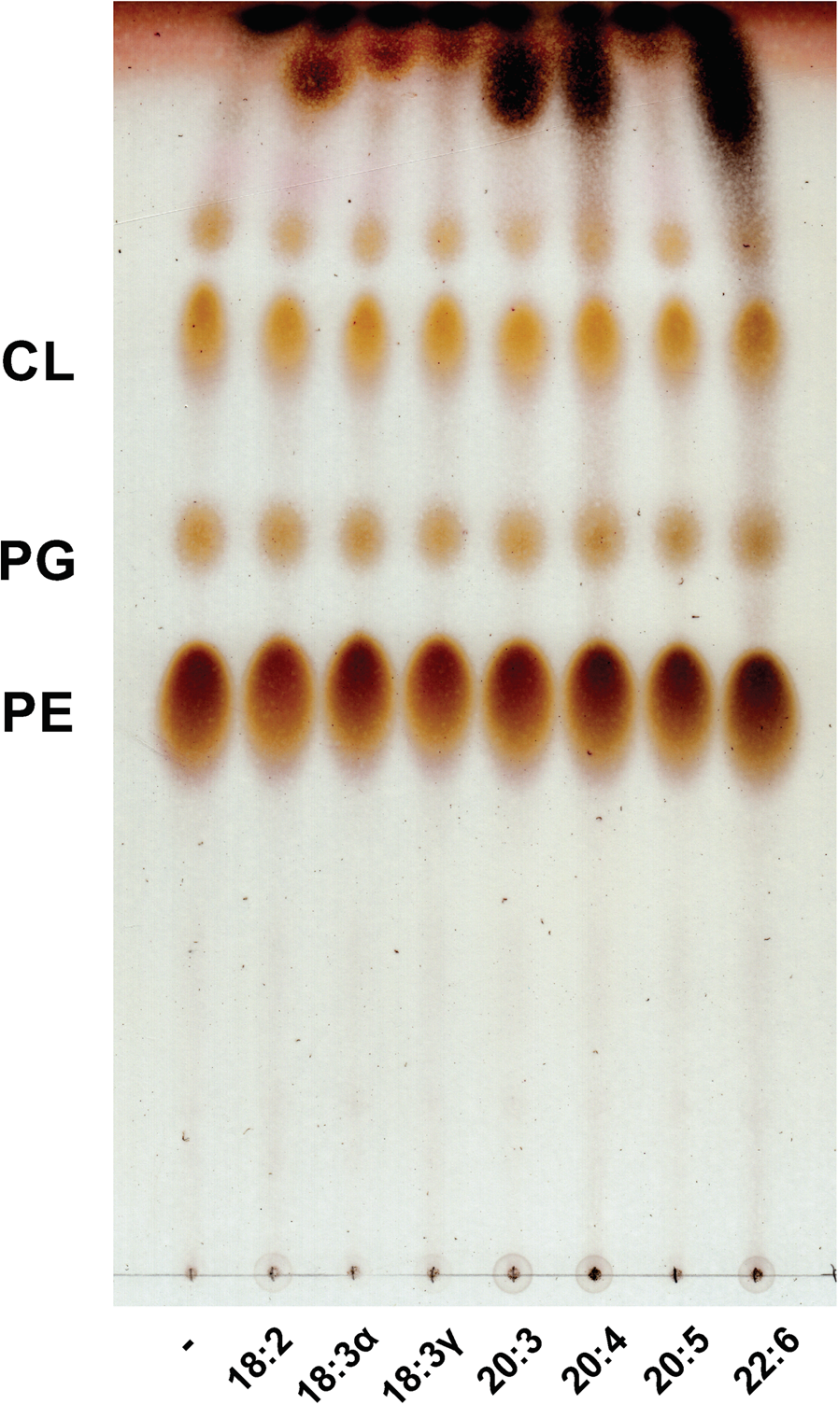
Thin-layer chromatography of phospholipids extracted from *E. coli* following growth with exogenous PUFAs. Bacteria were grown to exponential phase (OD ≈ 0.8) in CM9 media at 37 °C with or without 300 μM of the indicated fatty acids (linoleic acid [18:2], alpha-linoleic acid [18:3α], gamma-linolenic acid [18:3γ], dihomo-gamma-linolenic acid [20:3], arachidonic acid [20:4], eicosapentaenoic acid [20:5] and docosahexaenoic acid [22:6]) prior to Bligh and Dyer extraction of phospholipids and separation by TLC in the solvent system chloroform/methanol/acetic acid (65:25:10 v/v/v). The plate was charred and scanned to produce the final image

The membrane phospholipid incorporation of exogenous fatty acids was analyzed first by thin-layer chromatography of extracted phospholipids from *E. coli* grown to logarithmic phase in the presence of 300 μM of each fatty acid. The migration of the major bacterial phospholipids phosphatidylethanolamine (PE), phosphatidyglycerol (PG), and cardiolipin (CL) provided little, if any, qualitative data regarding incorporation of PUFAs (Fig. 2). The spots at the top of the plate reflect free fatty acids that were not washed from the bacteria during the Bligh and Dyer extraction and thus represent fatty acids associated with the bacteria and/or imported but not (yet) assimilated.

For confirmation of fatty acid incorporation into *E. coli* phospholipids, negative ionization UPLC/ESI-MS was performed on total lipid extracts. The 50 V sampling cone of the mass spectrometer is of sufficient energy to cause minor in-source fragmentation, thus allowing simultaneous observation of [M – H]^−^ parent ions and their cone fragments in the mass spectra of each chromatographically resolved component. Since the most prominent cone fragments of phospholipids consist of the carboxylate substituents attached to sn-1 and sn-2 positions of the glycerol backbone (Hsu and Turk 2000; Hsu and Turk 2001), in-source cone fragmentation allows unambiguous determination of phospholipid composition by directly observing the incorporated fatty acid residues.

**Fig. 3:**
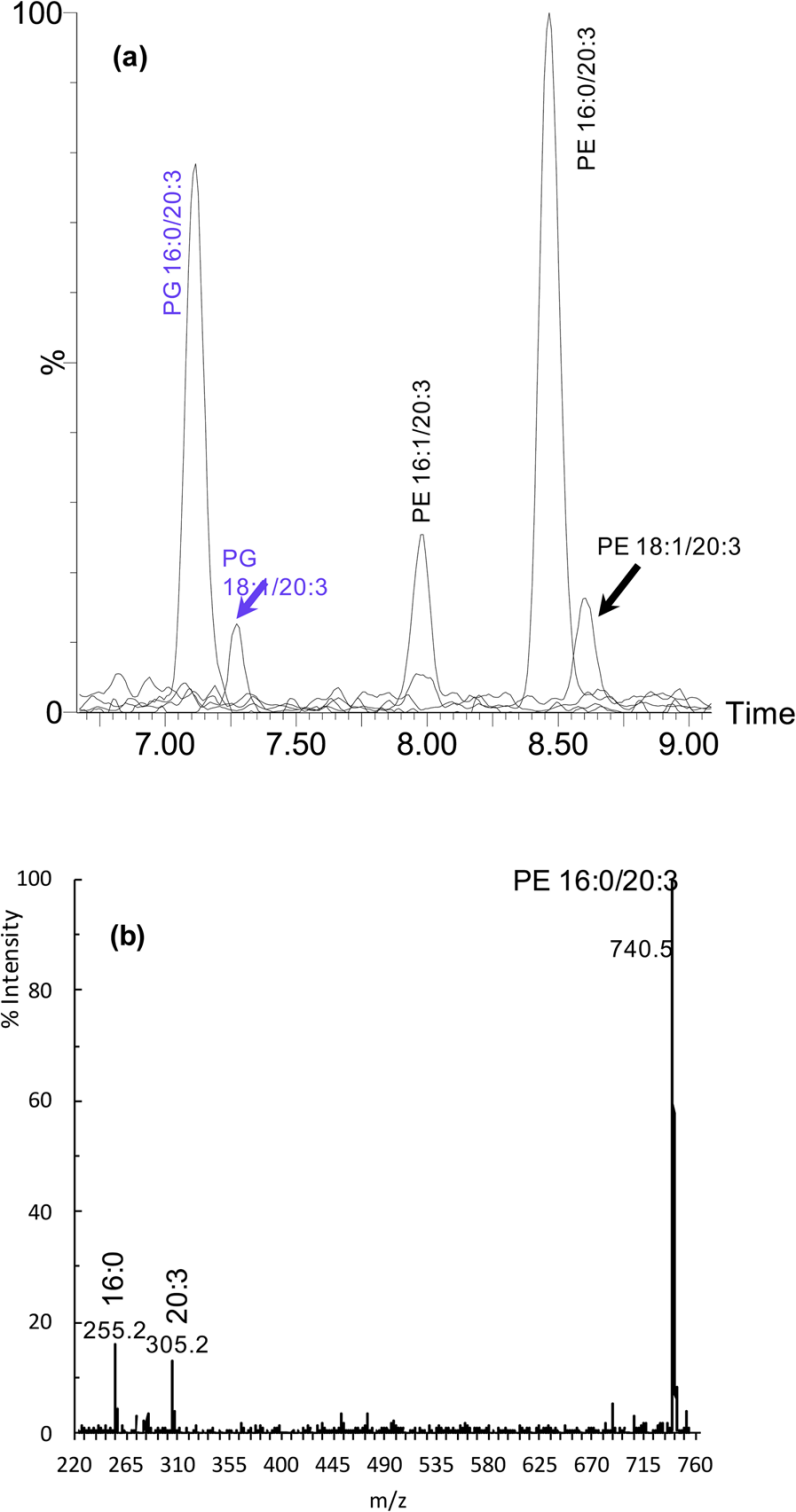
UPLC-MS of phospholipids extracted from *E. coli* grown in the presence of 20:3 fatty acid. [M-H]^−^ ions were detected by quadrupole mass spectrometry following electrospray ionization. **a** Overlain extracted ion chromatograms corresponding to the [M-H]^−^ ions of the indicated parent phospholipid. Note that the chromatographic gradient fully resolves species that differ by one double-bond; the extra double-bond increases the polarity such that PE (16:1/20:3) elutes 0.5 min earlier than PE (16:0/20:3). The control culture was analyzed for all of these same peaks, but none were detected. **b** Mass spectrum of the large peak at 8.5 min. The singly charged parent ion at 740.5 m/z has mass consistent with a PE (36:3) species. Direct observation of the cone fragments at 255.2 and 305.2 m/z, corresponding to 16:0 and 20:3 carboxylate ions respectively, confirm that the parent phospholipid is PE (16:0/20:3). Thus, it is confirmed that cultures grown in the presence of 20:3 fatty acid are capable of incorporating the supplemented PUFA into the membrane phospholipids. All other PUFAs tested, except 22:6, were likewise observed to be incorporated (Supplemental Figure 1)

All PUFAs, with the exception of 22:6, were identified as being incorporated at the sn2 position of PE and PG. For example, phospholipids extracted from cell cultures grown in the presence of 20:3 (Fig. 3) had chromatographic signals corresponding to PG (16:0/20:3), PG (18:1/20:3), PE (16:1/20:3), PE (16:0/20:3), and PE (18:1/20:3), whereas the control did not show such signals. The relative incorporation of all PUFAs, except 22:6, is portrayed in Supplemental Fig. 1.

### Membrane permeability is altered by exogenous fatty acids

**Fig. 4:**
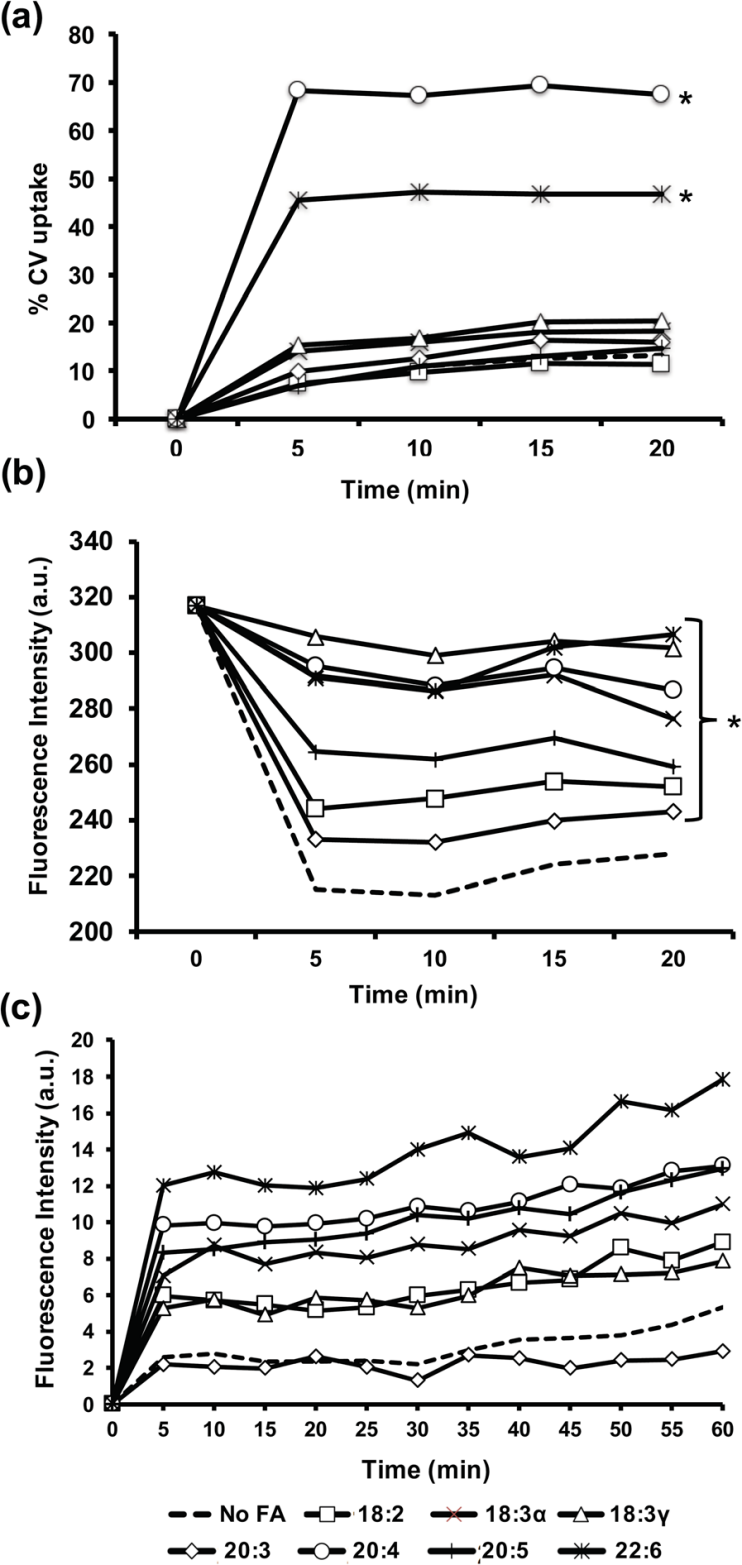
The effect of exogenous PUFAs on hydrophobic compound uptake in *E. coli*. **a** Bacteria were grown at 37 °C in CM9 with and without 300 μM of the indicated fatty acids to mid-log phase (OD = 0.8). Cultures were gently pelleted, washed with PBS and resuspended in an equal volume of PBS (OD_600_ = 0.6). The amount of CV in the supernatant following centrifugation was measured at regular intervals and expressed graphically as percentage of CV uptake. All standard deviations were less than 4% (not graphed for visual clarity). **b** Bacteria were grown at 37 °C in CM9 with and without 300 μM of the indicated fatty acids to mid-log phase (OD = 0.8). Cultures were gently pelleted, washed with PBS and resuspended in an equal volume of PBS (OD_600_ = 0.7). The amount of EtBr in the supernatant following centrifugation was measured as fluorescence emission intensity at 585 nm (excitation wavelength of 530 nm). Asterisks indicate significant difference (*,*p* < 0.002) as compared to control. **c** Bacteria were grown at 37 °C in CM9 with and without 300 μM of the indicated fatty acids to mid-log phase (OD = 0.8). Cultures were gently pelleted, washed with PBS and resuspended in an equal volume of PBS (OD_600_ = 0.4). Following addition of 20 μM EtBr, fluorescence intensity was measured every 5 m for 1 h (excitation wavelength of 545 nm; emission wavelength of 600 nm)

To evaluate the ramifications of newly adopted phospholipid species in the *E. coli* membrane, we next examined the effect on membrane permeability. First, a crystal violet uptake assay indicated that arachidonic acid and docosahexaenoic acid elicited significant increases (50 and 25%, respectively) in permeability as compared to the control and other fatty acids (Fig. 4a). A second evaluation of permeability was performed using ethidium bromide (EtBr) to assess both uptake and accumulation over time. The uptake assay (Fig. 4b) largely correlated with the accumulation assay (Fig. 4c), which measures the fluorescence intensity of live cells (increasing as EtBr binds to DNA). All PUFAs increased membrane permeability. The only fatty acids to display a different trend were 18:3γ and 20:3, which excluded EtBr yet displayed lower accumulation (Fig. 4b&c). Despite the differential uptake between dyes, these assays illustrate the varied and significant impact of exogenous fatty acids on membrane permeability.

### Exogenous fatty acids impact antimicrobial peptide resistance

**Fig. 5:**
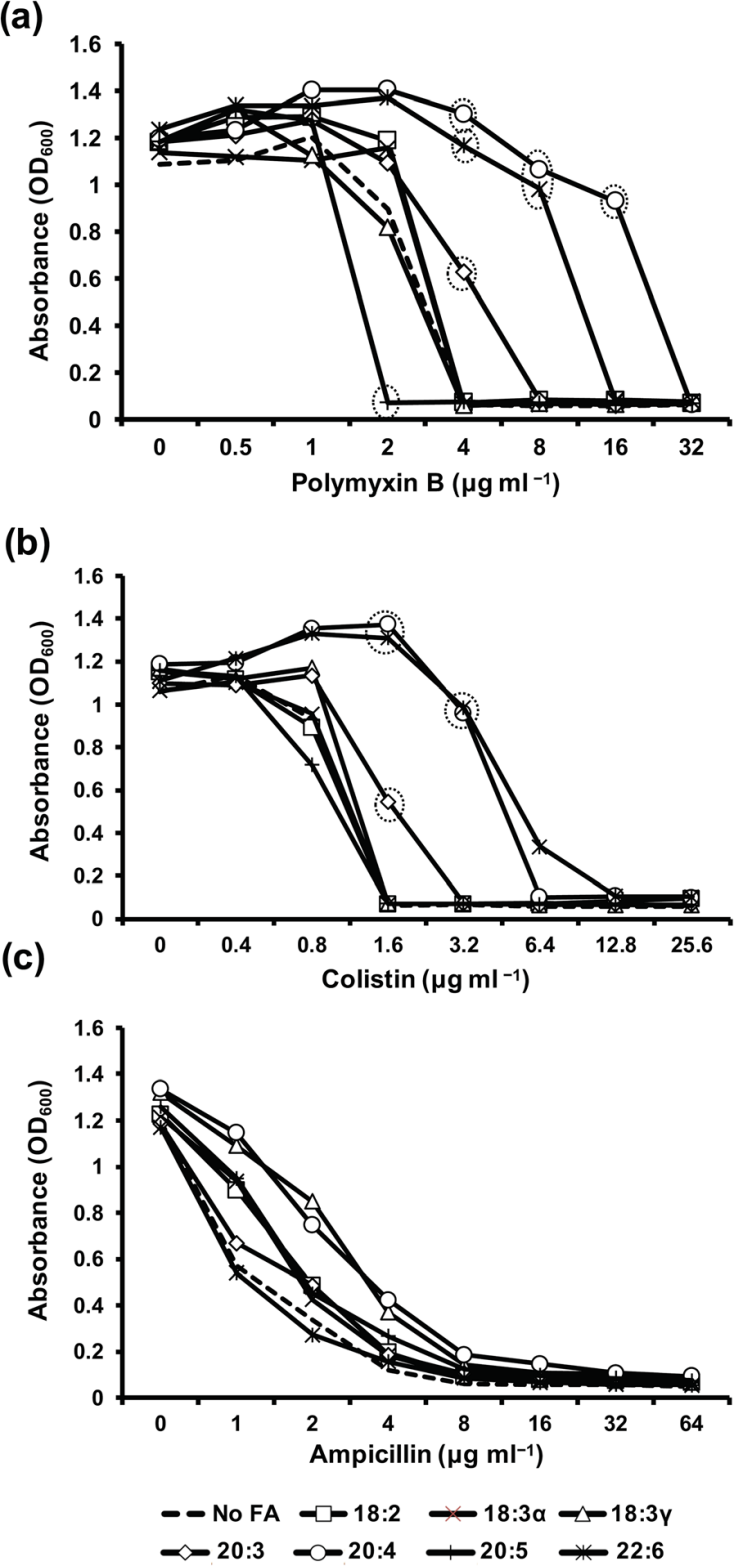
The effect of exogenous fatty acids on polymyxin B, colistin, and ampicillin resistance in *E. coli*. Bacteria were grown at 37 °C in CM9 with and without 300 μM of the indicated fatty acids to mid-log phase (OD = 0.8). Cultures were pelleted, washed with CM9 and resuspended in CM9 to an OD_600_ of 0.12. Fatty acids were again added to a final concentration of 300uM. The bacterial suspension was distributed into microtiter plates and two-fold concentrations of polymyxin B, colistin, or ampicillin were added. After 20 h incubation at 37 °C, the optical density (600 nm) was read using a Biotek Synergy microplate reader. Shown is a representative from two independent experiments conducted in triplicate, with each value representing the mean (all standard deviations < 0.04). Symbols circled by dotted line indicate significant differences (*p* < 0.002) as compared to the control (no fatty acid) at the particular antimicrobial concentration

The observed alteration to membrane permeability led to an investigation of the impact of fatty acids on antimicrobial peptide susceptibility, a phenomenon previously documented for several Gram-negative bacteria [1–4]. For these experiments, two antimicrobials (polymyxin B and colistin) with mechanisms of action that target membrane bilayers via biophysical intercalation were chosen to compare with an antibiotic (ampicillin) that relies on protein-mediated uptake for activity. In the minimum inhibitory concentration (MIC) assays, bacteria were pre-adapted with fatty acids prior to the assay, where fatty acids were also made available at 300 μM during exposure to two-fold concentrations of each antimicrobial. The availability of three fatty acids (20:3, 20:4, and 22:6) increase the MIC of *E. coli* against polymyxin B and colistin (Fig. 5a&b). Strikingly, arachidonic acid raised the MIC to polymyxin B 8-fold compared to the no fatty acid control. Eicosapentaenoic acid (20:5) was the only fatty acid to lower the MIC to polymyxin B. Both arachidonic acid and docosahexaenoic acid increased the MIC to colistin by 4-fold. Minimal differences were observed for the MIC of ampicillin, although 20:4 and 18:3γ-treated cultures maintained better survival at lower antibiotic concentrations (Fig. 5c).

### Exogenous PUFAs affect phenotypes associated with virulence

**Fig. 6:**
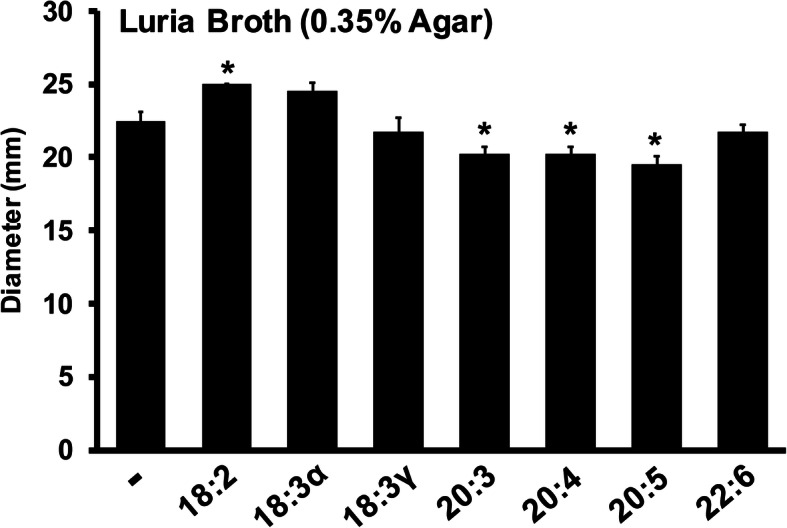
The effect of exogenous PUFAs on motility of *E. coli*. Soft agar motility plates were prepared, supplemented with 300 μM of the appropriate fatty acid or absence thereof (after cooling to 55 °C). 1 μL of inoculum (OD_600_ = 0.1) was pipetted into motility plates and observed after 12 h incubation at 30 °C. Each value represents the mean and standard deviation. Shown is a representative assay from two separate biological replicates performed in quadruplicate. Asterisks indicate *p*-values determined to be less than 0.005 when compared to the no fatty acid controls

**Fig. 7:**
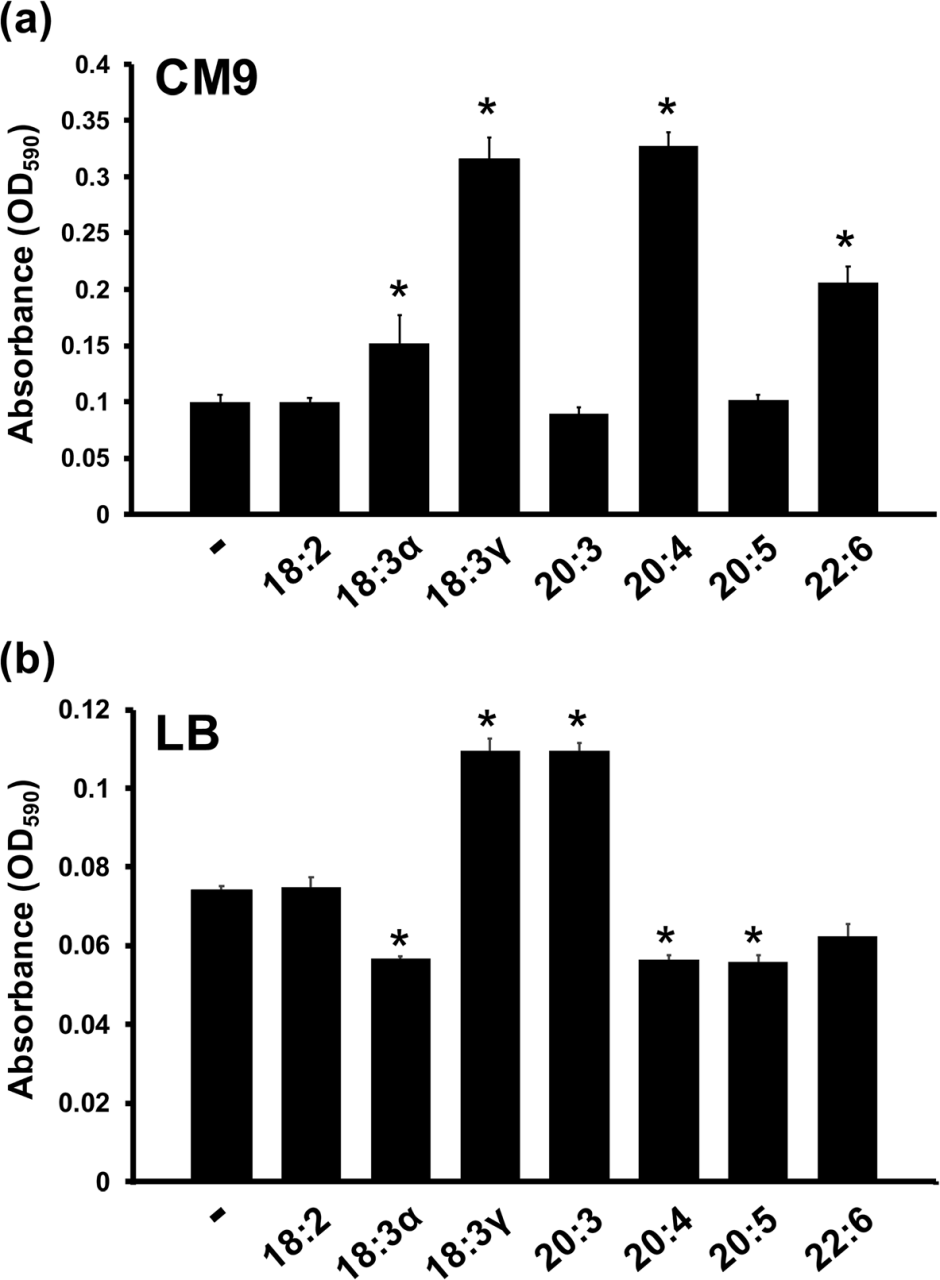
Incubation with PUFAs alters biofilm formation in *E. coli*. Overnight cultures were pelleted, washed, resuspended in appropriate media and inoculated onto microtiter plates (starting OD ~ 0.1) in octuplet. Each culture was grown in the presence of 300 μM of the indicated fatty acids. After 24 h incubation, the biofilm assay by O’Toole was performed in complex (LB) and minimal media (CM9). The absorbance (OD_590_) was measured using a Biotek Synergy microplate reader. Shown is a representative assay from two independent experiments. Each value represents the mean and standard deviation of 8 wells. Asterisks indicate *p*-values determined to be less than 0.001 when compared to the no fatty acid controls

Bacterial motility and biofilm formation are phenotypes known to influence pathogenesis. Since previous studies have identified fatty acid-induced alterations in other Gram-negative bacteria for these phenotypes, this study also investigated swimming motility and biofilm formation in *E. coli*. The availability of 300 μM fatty acid in a soft agar assay resulted in increased motility (≈10%) with 18:2 and decreased motility (≈10%) for the 20-carbon fatty acids tested (20:3, 20:4, 20:5) (Fig. 6). More striking differences were observed for biofilm formation, where supplementation of 18:3α, 18:3γ, 20:4, and 22:6 significantly increased the amount of biofilm when the assay was performed in CM9 minimal media (Fig. 7). In particular, biofilm formation was doubled with 22:6 and tripled with 18:3γ and 20:4. When the assay was performed in LB, 18:3γ and 20:3 elicited significant increases in biofilm formation, whereas 18:3α, 20:4, and 20:5 decreased biofilm formation to a near identical degree. Collectively, the assessment of phenotypes associated with virulence revealed several exogenous fatty acid-mediated impacts on swimming motility and biofilm formation.

## Discussion

A new paradigm of Gram-negative bacterial utilization of fatty acids has been emerging over the past decade. No longer are fatty acids merely a carbon source destined for β-oxidation; they represent multipurpose molecules capable of nutritional value, membrane phospholipid remodeling, and signal-mediated behavioral control. Previous studies from our laboratory have explored these phenomena in several medically important bacteria, including *Vibrio* species, *Pseudomonas aeruginosa*, *Klebsiella pneumoniae*, and *Acinetobacter baumanii* [1–4]; however, an analysis of the model organism *E. coli* had not been performed.

The current study examined the handling of exogenous fatty acids in *E. coli* more than thirty years after the discovery of the outer membrane fatty acid transporter FadL. We report assimilation of several PUFAs into membrane phospholipids as determined by UPLC/MS. PUFA incorporation altered membrane permeability when assessed using two hydrophobic dyes. All PUFAs enhanced growth into stationary phase, while most PUFAs supported growth as sole carbon sources. Significant changes to the MICs of polymyxin B and colistin were observed for some fatty acids, while sensitivity to ampicillin was largely unaffected. Finally, two phenotypes associated with virulence were examined for responsiveness to exogenous fatty acids, with motility yielding modest effects and biofilm formation showing significant fluctuations depending on media and supplied PUFA.

As expected, the supplementation of individual fatty acids into growth medium resulted in elevated cell density compared to the control devoid of fatty acids, an effect observed as early as 3 h and further supported by CFU depermination at 7 h. The growth phenotype is attributed to fatty acids serving as extra carbon sources available for bacterial metabolic acceleration. Interestingly, an evaluation of fatty acids as sole carbon sources identified three fatty acids (18:2, 20:3, and 22.6) that elicited higher cell density over the course of 12 h. Furthermore, determination of CFU at hour 7 supported the substantial growth of 18:2-fed cultures, as well as nominal growth/persistence of bacteria supplemented with other PUFAs. A more comprehensive study would be necessary for determining the relative preferences and growth consequences during incubation with PUFAs as sole carbon sources.

The thin-layer chromatography analysis of phospholipids did not provide convincing qualitative evidence for incorporation of exogenous fatty acids (Fig. 2). Compared to other similarly performed analyses from our laboratory [1–4], the migratory shifts were much less pronounced, suggesting a lower degree of PUFA incorporation. Subsequent UPLC/MS analyses confirmed at least some degree of incorporation of all fatty acids except 22:6 into PE and PG. The exogenously provided fatty acids were detected via mass spectrometry as their respective carboxylate ions cleaved from either PE or PG using in-source cone voltage induced fragmentation. Ongoing studies aim to better characterize the underlying mechanisms involved in PUFA uptake and incorporation using mutant strains (eg, *fadL*, *fadD*, and *pls*), as well as quantitative assessment to compare PUFA incorporation between Gram-negative bacteria.

A survey of the membrane permeability consequences yielded contrasting results depending on hydrophobic dye used in the assay. The uptake assay utilizing crystal violet indicated significantly increased permeability when arachidonic acid and docosahexaenoic acid were available. Similarly, the uptake and accumulation assays with ethidium bromide indicated that 20:4 and 22:6 caused the most elevated permeability (Fig. 4). Dihomo-gamma-linolenic acid (20:3) elicited minimal change to permeability. Few trends were identified with regard to stereochemistry (omega-3 vs. omega-6), carbon chain length, or degree of unsaturation. In the ethidium bromide influx assay, the fatty acids with longest carbon length and highest unsaturation corresponded to the highest observable accumulation (Fig. 4). Here we performed a cursory examination of overall permeability using hydrophobic dyes; thus, the possible role of efflux was not considered. Indeed, exogenous fatty acid-mediated alterations to Gram-negative membrane permeability warrant analyses in future studies.

Considering the influence of PUFAs on membrane permeability, we next considered the ramifications of antimicrobial activity using membrane active antibiotics (polymyxin B and colistin). Strikingly, the availability of several exogenous fatty acids drastically changed the MICs of polymyxin B and colistin. While many studies have attributed altered antimicrobial peptide MICs to LPS modifications [18, 19], a few studies have identified phospholipid amount and composition as a contributing factor [20, 21]. Developing antimicrobials that target the LPS synthesis and modification pathways are (and should be) primary goals; however, phospholipids represent another potential avenue for identifying vulnerabilities in bacterial membranes. Since antimicrobial peptides capitalize on membrane charge and bilayer intercalation to create bacterial membrane vulnerabilities, the assimilation of exogenous fatty acids presents an interesting scenario that could heighten sensitivity to a spectrum of antimicrobials that possess mechanism of actions independent of protein-mediated passage through microbial membranes.

Despite significant alteration of both membrane permeability and antimicrobial resistance, 22:6 was not detected by UPLC/MS to be incorporated into membrane phospholipids. The likely explanation involves intercalation of the PUFA at the outer membrane, facilitating the passage of hydrophobic dyes and antimicrobials. A similar effect has been observed for the activity of lysozyme against *P. aeruginosa*. Using surface plasmon resonance and calorimetric analyses, 22:6 was responsible for initial bacterial membrane penetration, creating opportunity for further influx [22]. This observation introduces potential for dual effects of exogenous fatty acids on Gram-negative membranes that could influence permeability and antimicrobial susceptibility assessments: 1) fatty acid uptake and assimilation into phospholipids and/or 2) prerequisite PUFA-mediated membrane perturbation. Previous studies in our laboratory had not identified an unincorporated PUFA in Gram-negative bacteria; thus, the activity of 22:6 on *E. coli* necessitates reevaluation of the relative contributions of microbially-mediated versus chemically-mediated permeabilization.

Finally, an evaluation of phenotypes associated with virulence revealed minimal fatty acid-mediated effects (≈10%) on swimming motility for 18:2, 20:3, 20:4, and 20:5. While some of these responses mirror previous observation with other Gram-negative bacteria, a more rigorous examination of motility is warranted, including a survey of other modes of motility (swarming, surfing). The impacts on biofilm formation were diverse and more pronounced in minimal media, with strong increases observed upon supplementation with 18:3γ, 20:4, and 22:6. Biofilm formation was tested in a rich media (containing PUFAs) and a minimal media with individual PUFA supplementation for comparison. It was expected that the effect of a given PUFA would be magnified in the minimal media devoid of fatty acids. Indeed, more robust biofilm formation, along with stronger overall responses, was associated with minimal media. Although mechanistically uncharacterized, it is interesting to consider the potential ramifications of fatty acid utilization in vivo. For example, the importance of arachidonic acid for initiating inflammatory processes, such as lipid-mediated PMN chemotaxis during enteroaggregative *E. coli* infection [23], may represent an opportunity for pathogen-directed hijacking of host fatty acids to modulate virulence. In contrast, the experimental administration of PUFAs has inhibited pathogenesis in mouse models of infection for a variety of Gram-negative pathogens [24–27]. Yet another important aspect of PUFA membrane incorporation involves the ramification on membrane potential that may influence antibiotic sensitivity, among other electrophysiological processes.

## Conclusions

The data herein reinforce the growing body of literature highlighting polyunsaturated fatty acids as important resources for Gram-negative bacteria. These findings identify new and expanded roles of fatty acids in the lifestyle of the model organism *Escherichia coli*. Not only are uptake and incorporation of exogenous fatty acids confirmed and membrane permeability variably affected, but behavioral characteristics such as growth, motility and biofilm formation are influenced when PUFAs are available. Importantly, the presence of certain PUFAs altered the MICs of polymyxin B and colistin, suggesting a potential role of fatty acids in not only the pathogenesis of *E. coli* infections, but also prospective synergistic treatment avenues involving PUFA/antimicrobial combinations.

## Methods

### Bacterial strains and media

*Escherichia coli* MG1655, purchased from ATCC, was used for all experiments. Overnight cultures grown in Luria broth were pelleted, washed with PBS, and transferred to M9 minimal medium [0.4% glucose supplemented with 150 mM NaCl] for initiation of most experiments. All experiments were performed at 37 °C. Fatty acids used in this study were purchased from Cayman Chemicals [linoleic acid (18:2), α-linolenic acid (18:3α), γ-linolenic acid (18:3γ), dihomo-γ-linolenic acid (20:3), arachidonic acid (20:4), eicosapentaenoic acid (20:5), and docosahexaenoic acid (22:6)].

### Growth curves and CFU determination

Two growth curves were performed for *E. coli* grown in the presence of fatty acids. To assess growth response to fatty acid supplementation, bacteria were grown in M9 media supplemented with 0.4% casamino acids (CM9) in the presence and absence of 300uM fatty acid. To examine the response of *E. coli* with fatty acids as the sole carbon source, 1 mM fatty acid was supplemented. Both growth curves were conducted three times for a period of 12 h, and absorbance (OD_600nm_) was measured every hour. For colony forming unit (CFU) determination, serial dilutions were performed at hour 7 for each sample. Multiple dilutions were plated in triplicate and incubated overnight prior to colony counting. Plates containing 5–300 colonies were included for calculation of CFU/ml. Values represent average CFU/ml from at least 6 plates per sample.

### Phospholipid extraction

Lipids were extracted from bacterial cultures by following the Bligh and Dyer method [28]. Briefly, cultures were grown to OD_600_ = 0.8–0.9 prior to cell harvesting by centrifugation and solvent-mediated extraction of phospholipids. For thin layer chromatography (TLC), 14 mL of bacterial culture were used. For UPLC/MS, 20 mL cultures were used and an extra wash step was included prior to the final extraction. Total lipid extracts were dried under a stream of high-purity nitrogen gas and stored at − 20 °C for TLC or UPLC/MS analysis within one week.

### Thin layer chromatography

Lipids were separated on silica coated plates using a solvent system consisting of chloroform, methanol and acetic acid (65:25:10, v/v). The TLC plates were sprayed with 10% sulfuric acid in 100% ethanol and visualized by charring (heating) at 180 °C for approximately 30 s. The plate was cooled and immediately imaged using a Canon CanoScan 9000F.

### Ultra performance liquid chromatography-mass spectrometry

Liquid chromatography mobile phase solvents are defined as A1 (30:70 25 mM ammonium acetate: MeOH, pH 6.7), and B1 (pure MeOH). All solvents and additives used were Optima grade (Fischer Scientific). Samples for LC-MS analysis were prepared at 400 ppm (total lipid extract) in a 50:50 mixture of solvents A1 and B1. Analyses were performed using a Waters Acquity UPLC interfaced with a Quattro Micro quadrupole mass spectrometer using electrospray ionization in the negative mode (ESI-). Samples were loaded into the room temperature autosampler and 5 uL injected for gradient elution using an Acquity BEH C8 column (2.1 × 100 mm, 1.7 μm particles). The following gradient was used for analyte separation at a constant flow rate of 0.3 mL/min: 50:50 A1:B1 held constant for 2 min, a linear increase of solvent B1 over the course of 8 min, ultimately reaching 100% B1 at the 10 min mark, followed by a rapid decrease in B1 over the course of 0.3 min, resulting in the reestablishment of 50:50 A1:B1 by 10.3 min, which was held constant for an additional 0.7 min for a total run time of 11 min. The chromatographically separated analytes were eluted into the mass spectrometer and ionized using an electrospray capillary voltage of 1.5 kV to generate negative ions. Desolvation utilized 350 °C nitrogen gas flowing at 750 L/h with a source temperature of 140 °C. Analytes were introduced into the mass analyzer through a 50 V sampling cone, which is of sufficient energy to cause minor in-source cone fragmentation of the phospholipids. The minor fragmentation allows simultaneous observation of the intact phospholipid in addition to the attached fatty acid chains which are cleaved from the sn1 and sn2 positions as their respective carboxylate ions. Analysis of the *m/z* values for the [M - H]^−^ signals of the parent ion and the two cone-fragment carboxylate ions allows unambiguous identification of each eluted phospholipid. The quadrupole scan range was set to 200–1500 *m/z* with a scan time of 0.5 s and an interscan delay of 0.05 s. Resultant chromatograms and their corresponding mass spectra were analyzed using MassLynx v4.1 software.

### Motility assay

Soft agar, swimming motility assays were carried out in quadruplicate by adhering to the following protocol: Soft agar was prepared with 10 g L^− 1^ tryptone, 10 g L^− 1^ NaCl, 0.35% agar. Following autoclave sterilization and cooling to 55 °C, 40 mL of molten agar was added to 50 mL conical tubes containing one of each PUFAs at a final concentration of 300 μM. The plates were inoculated in quadruplicate with 2 μL of a bacterial suspension at OD_600_ of 1.0 in PBS. The plates were then incubated at 30 °C for a period of 12 h. Bacterial motility was assessed by measuring the diameter of the motility halo in each quadrant. Data represents two independent experiments and statistical analysis was carried out by using student’s t-test (paired, two-tailed, *p* < 0.01).

### Permeability assays

Three different membrane permeability assays were carried out to determine the effects of phospholipid remodeling in *E. coli* by following previously established procedures [2, 3]. The first assay monitored bacterial uptake of the hydrophobic compound crystal violet (CV). Eight separate *E. coli* cultures were grown to OD_600_ 0.9 ± 0.05 in the presence (or absence) of one of the seven PUFAs at a concentration of 300 μM in CM9. The cultures were pelleted, washed with PBS, and resuspended in 5 mL of PBS to OD 0.4. Crystal violet was then added to each of the cell cultures at a concentration of 5 μg/mL. Absorbance measurements were made in five-minute intervals (for a total of 20 min) by pelleting 1 mL of cell culture via centrifugation, decanting the supernatant, and collecting absorbance readings (590 nm). Data was converted to percentage of CV taken up based on a control tube that contained PBS and CV but no bacteria to estimate maximal CV. Two ethidium bromide assays were performed as previously described [1], one measuring uptake and the other measuring accumulation. The uptake assay protocol was identical to the CV assay. Ethidium bromide assays were measured using a Varian Cary Eclipse Fluorescence Spectrophotometer with appropriate excitation and detection wavelengths. Three biological replicates were performed for the uptake assays and two biological replicates were performed for the accumulation assay.

### Antimicrobial peptide susceptibility assay

A previously established protocol was used to determine the effect that PUFA exposure has on antimicrobial peptide susceptibility in *E. coli* [2, 4]. Cultures were grown to logarithmic phase in CM9 minimal medium in the presence or absence of one of the seven PUFAs at a concentration of 300 μM. Following centrifugation, the cultures were washed with CM9 minimal medium and resuspended at an OD_600_ 0.12and supplemented with fatty acid to yield a final concentration of 300 μM. A volume of 170 μL of each *E. coli* culture was added to the wells of a 96-well microtiter plate containing 30 μL of a two-fold concentration of each antimicrobial peptide (polymyxin B and colistin). Following an incubation period of 24 h at 37 °C, absorbance measurements were made at 600 nm using a Biotek Synergy microplate reader. Two biological replicates were performed in triplicate for each antimicrobial peptide.

### Biofilm formation assay

A previously published protocol for microtiter plate assessment of biofilm formation was used [29]. Overnight *E. coli* cultures grown in LB were pelleted, washed and resuspended in CM9 (M9 minimal medium supplemented with casamino acids) containing one of the seven PUFAs at a concentration of 300 μM prior to incubation at 37 °C for 24 h. Planktonic cells were removed and the plates were washed three times with deionized water. Biofilms were stained with 3% crystal violet solution, incubated at room temperature for 15 min, and washed three times with deionized water. After air drying, 30% acetic acid solution was added to each well and incubated for 15 min. The dissolved crystal violet was transferred to a fresh 96-well microtiter plate, and absorbance measurements were made for each well at 590 nm using a Biotek Synergy microplate reader. The assay was carried out twice in octuplet and statistical analysis was performed using student’s t-test (paired, 2-tailed, *p* < 0.001).

## Supplementary information

**Additional file 1.**

## Data Availability

The datasets during and/or analysed during the current study available from the corresponding author on reasonable request.

## Abbreviations

PUFA: polyunsaturated fatty acid
PG: phosphatidylglycerol
PE: phosphatidylethanolamine
CL: cardiolin (diphosphatidylglycerol)
