# Supplementary material for: Exogenous polyunsaturated fatty acids (PUFAs) promote changes in growth, phospholipid composition, membrane permeability and virulence phenotypes in Escherichia coli
Source: BMC Microbiol. 2020 Oct 12;20:305. doi: 10.1186/s12866-020-01988-0 (PMC7552566; doi:10.1186/s12866-020-01988-0)
Supplement: Supplementary file 1 — Additional file 1. [file 12866_2020_1988_MOESM1_ESM.zip › Supplemental Figure 1_ESM.docx]

**PE 16:0/18:2**

[M-H]^–^

m/z 714

279

255

(c)

[M-H]^–^

m/z 712

277

**PE 16:0/18:3α**

255

(b)


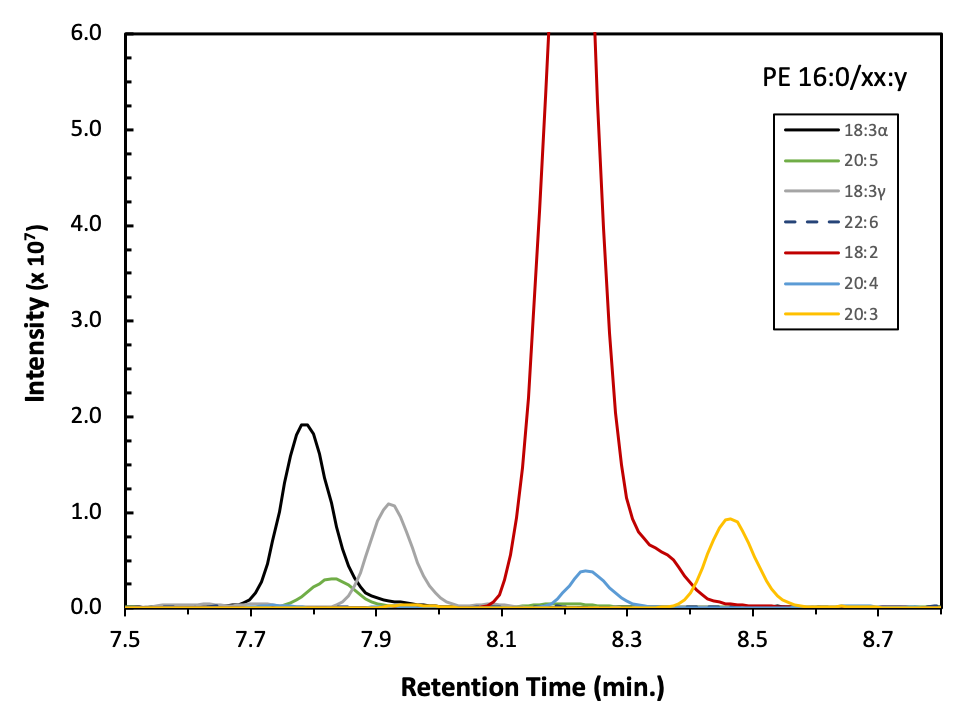


(a)

Supplementary Figure 1.

LC-MS analysis of total lipid extracts from *E. coli* grown in the presence of various PUFAs. Each PUFA corresponds to separate cultures, but all have been overlain onto one figure for comparison. (a) Extracted ion chromatograms of [M-H]^–^ ions showing incorporation of all PUFAs, except 22:6, into the phosphatidylethanolamine structure in which sn-1 is occupied by 16:0. (b) High cone voltage (50 V) mass spectrum with parent ion [M-H]^–^ at *m/z* 712 and cone fragments at *m/z* 255 and 277 corresponding to PE 16:0/18:3α eluted at 7.8 min. (c) High cone voltage (50 V) mass spectrum with parent ion [M-H]^–^ at *m/z* 714 and cone fragments at *m/z* 255 and 279 corresponding to PE 16:0/18:2 eluted at 8.2 min.
