# Supplementary material for: Exogenous polyunsaturated fatty acids (PUFAs) promote changes in growth, phospholipid composition, membrane permeability and virulence phenotypes in Escherichia coli
Source: BMC Microbiol. 2020 Oct 12;20:305. doi: 10.1186/s12866-020-01988-0 (PMC7552566; doi:10.1186/s12866-020-01988-0)
Supplement: Supplementary file 1 — Additional file 1. [file 12866_2020_1988_MOESM1_ESM.zip › Supplemental Table 1_ESM.docx]

**Supplemental Table 1.**

**Growth Curve (Figure 1A)**

|  | **No FA** | **18:2** | **18:3α** | **18:3γ** | **20:3** | **20:4** | **20:5** | **22:6** |
| --- | --- | --- | --- | --- | --- | --- | --- | --- |
| **CFU/ml**  **at hour 7** | 2.8e9 | 3.2e9 | 3e9 | 2.9e9 | 3.2e9 | 2.8e9 | 2.7e9 | 2.9e9 |

All standard deviations < 2.7e8

|  | **No FA** | **18:2** | **18:3α** | **18:3γ** | **20:3** | **20:4** | **20:5** | **22:6** | **Glu** | **EtOH** |
| --- | --- | --- | --- | --- | --- | --- | --- | --- | --- | --- |
| **CFU/ml**  **at hour 7** | 4.5e7 | 1.1e8 | 7e7 | 5e7 | 5.5e7 | 5.5e7 | 5.5e7 | 6e7 | 1e9 | 4.4e7 |

**Sole Carbon Growth Curve (Figure 1B)**

All standard deviations < 8e6
